# Breast organoid suspension cultures maintain long-term estrogen receptor expression and responsiveness

**DOI:** 10.1038/s41523-024-00714-7

**Published:** 2024-12-19

**Authors:** Kung-Chi Chang, Francesca Silvestri, Michael U. J. Oliphant, M. Angie Martinez-Gakidis, Dennis P. Orgill, Judy E. Garber, Deborah D. Dillon, Joan S. Brugge

**Affiliations:** 1 Department of Cell Biology and Ludwig Center at Harvard, Harvard Medical School, 240 Longwood Ave, Boston, MA 02115 USA; 2 Department of Surgery, Division of Plastic and Reconstructive Surgery, Brigham & Women’s Hospital, 75 Francis St, Boston, MA 02115 USA; 3 Department of Medical Oncology, Dana-Farber Cancer Institute, 450 Brookline Ave, Boston, MA 02115 USA; 4 Department of Pathology, Brigham & Women’s Hospital, 75 Francis St, Boston, MA 02115 USA

**Keywords:** Hormone receptors, Breast cancer

## Abstract

Organoid cultures offer a powerful technology to investigate many different aspects of development, physiology, and pathology of diverse tissues. Unlike standard tissue culture of primary breast epithelial cells, breast organoids preserve the epithelial lineages and architecture of the normal tissue. However, existing organoid culture methods are tedious, difficult to scale, and do not robustly retain estrogen receptor (ER) expression and responsiveness in long-term culture. Here, we describe a modified culture method to generate and maintain organoids as suspension cultures in reconstituted basement membrane (™Matrigel). This method improves organoid growth and uniformity compared to the conventional Matrigel dome embedding method, while maintaining the fidelity of the three major epithelial lineages. Using this adopted method, we are able to culture and passage purified hormone sensing (HS) cells that retain ER responsiveness upon estrogen stimulation in long-term culture. This culture system presents a valuable platform to study the events involved in initiation and evolution of ER-positive breast cancer.

## Introduction

Breast cancer is a heterogeneous disease classified into distinct subtypes based on pathologic status of estrogen receptor (ER), progesterone receptor (PR), and human epidermal growth factor receptor 2 (HER2) combined with molecular examination of other markers^1–4^. Most breast cancers are ER-positive (about 70%) with or without co-expression of PR^5^. In contrast to normal breast, ER^+^ proliferating cells are common in luminal tumors and their proliferation is inhibited by ER-antagonists^1–4^, suggesting that the development of ER^+^ human breast cancer is associated with dysregulation of ER and proliferation. Investigations of the evolution of ER^+^ cells, the mechanisms of their transformation, and the effects of distinct genetic alterations in ER^+^ cells have been limited due to lack of culture conditions that maintain ER expression and estrogen responsiveness in breast epithelial cells.

The advent of organoid technology has enabled the development of normal breast organoid cultures that preserve the differentiated epithelial cell lineages of the native tissue: basal/myoepithelial (BA) cells, luminal adaptive secretory precursor (LASP) and hormone-sensing (HS) cells. These cultures have enabled the investigation of different stages of mammary gland development as well as characterization of the earliest changes in breast tissues of women at high risk of developing breast cancer^6,7^. However, existing organoid culture protocols, which involve embedding of cells in solid 100% reconstituted basement membrane (™Matrigel) domes, are tedious to passage, difficult to scale for high throughput studies, and generally do not robustly preserve ER expression and responsiveness over time. These limitations preclude the generation of organoid cultures from small amounts of tissue and the ability to study the evolution of ER^+^ tumor cells.

Here, we describe a modified normal breast organoid culture system that preserves the three breast epithelial lineages, increases organoid growth rate and uniformity, and maintains ERα expression and estrogen responsiveness in HS cells in long-term culture.

## Results

### Suspension culture produces larger and more uniform human breast organoids than conventional Matrigel domes

**Fig. 1:**
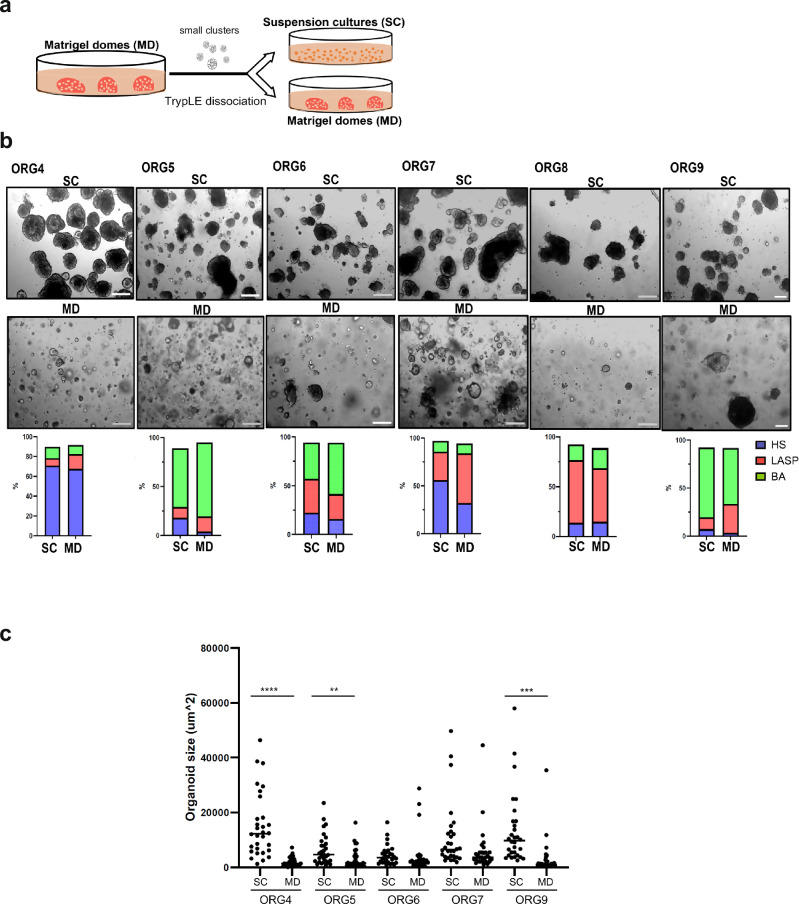
Propagation of normal human breast organoids as suspension cultures. **a** Established breast organoid cultures derived using the conventional Matrigel dome method (passage 5–6^14^) were dissociated and either re-cultured in Matrigel domes or as suspension cultures in 5% Matrigel for more than two months. **b** Representative brightfield images of six matched organoid lines grown as Matrigel domes (MD) or suspension cultures (SC) and their corresponding percentage of HS, LASP and BA cells based on FACS sorting using EpCAM and CD49f antibodies (4× magnification, scale bar 200 μm). **c** Mean organoid size of the indicated matched cultures grown as domes or in suspension. A total of 30 organoids per line per culture condition were measured using Image J. **p value < 0.01, ***p value < 0.001, ****p value < 0.0001, paired t-Test, two-tailed.

Several studies of cancer organoids have suggested that suspension cultures supplemented with a low percentage of Matrigel can not only be used as an alternative culture condition but also increase cancer organoid expansion rate compared to traditional cultures embedded in solid Matrigel domes^8–11^. To evaluate whether suspension culture could also shorten the expansion time of normal breast organoids, we first tested seven previously established organoid lines. These seven organoid lines were generated from normal breast tissues obtained from reduction mammoplasty or prophylactic mastectomy using conventional Matrigel dome cultures as previously described^6,12,13^. We then dissociated these organoids to small clusters (20–60 cells per organoid) and re-cultured half in solid Matrigel domes and the other half in ultra-low attachment plates with 5% Matrigel in the media and monitored growth and morphology of the organoids for more than two months (Fig. 1a, Supplementary Fig. 1a). As previously described, human breast organoids cultured in solid Matrigel domes are heterogeneous, forming various types of structures in the different organoid lines^6,13^. In contrast, organoids cultured in suspension were generally larger in size and exhibited a more uniform sphere-like structure (Fig. 1b, c, Supplementary Fig. 1b). Since we observed larger organoids in suspension cultures, we assessed the proportion of proliferating cells in ORG14 using two methods: EdU labeling and flow cytometry (see Methods) as well as Ki-67 immunofluorescence staining (Supplementary Fig. 1c, d). The ORG14 organoids in the suspension cultures contained a larger proportion of proliferating cells than organoids in Matrigel domes (Supplementary Fig. 1c–e). There was very little variation in organoid size for the dome cultures. Moreover, there was a correlation between the fraction of Ki-67^+^ cells and organoid size only in the suspension cultures (*r* = 0.57, Supplementary Fig. 1f), indicating that larger organoids contain more proliferating cells.

To assess the representation of breast epithelial lineages in the suspension organoid cultures, we performed flow cytometry using the canonical lineage markers EpCAM and CD49f to compare the proportion of HS (EpCAM^high^ CD49f^low^), LASP (EpCAM^high^ CD49f^high^), and BA (EpCAM^low^ CD49f^high^) cells to organoids cultured in solid Matrigel domes. These three major epithelial cell types were all maintained in the two culture conditions but, as previously reported^14^, their proportions varied in cultures from different donors. Overall, the proportions of the three cell types were roughly similar although four out of six organoid lines contained a higher HS proportion in the suspension cultures (Fig. 1b).

Next, we examined the morphology of the suspension organoids by performing immunofluorescence of established breast epithelial lineage markers: FOXA1 for HS, CD133 for LASP, and α-SMA for BA cells. In addition to validating the proportions we identified by flow cytometry, we found that all three epithelial cell types were present within most individual organoids and exhibited a uniform staining pattern, with luminal cells (HS and LASP cells) in the interior and BA cells distributed over the outer area of the organoids (Supplementary Fig. 1g), indicating that Matrigel suspension preserves the ability of normal breast epithelial cells to self-organize into acinar-like structures under these conditions. Previously, we had found that bilayered structures were lost with passage in dome cultures^12,14^.

### Breast organoids cultured in suspension contain a higher proportion of proliferating cells than those in Matrigel domes

**Fig. 2:**
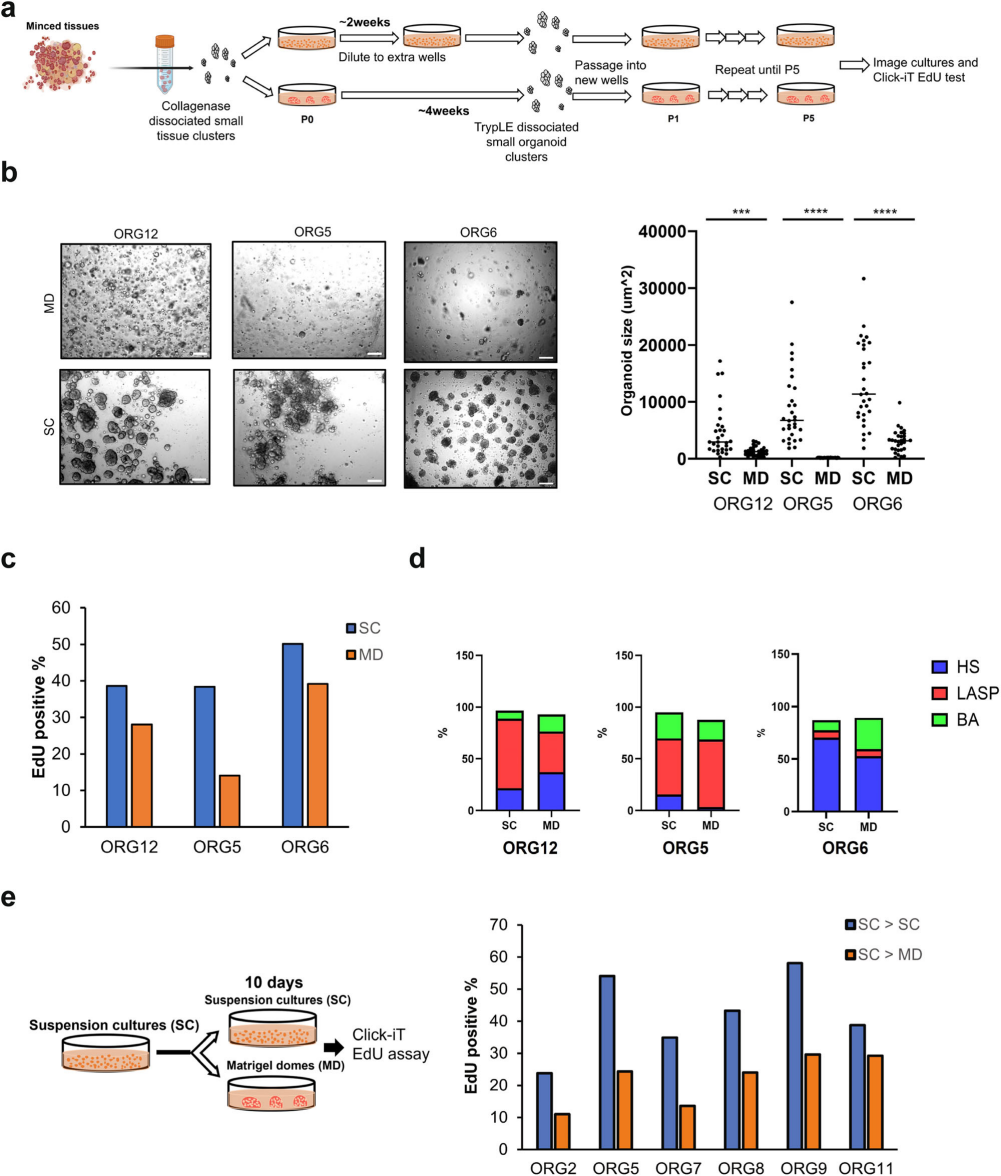
Breast organoid suspension cultures have a higher fraction of proliferating cells than Matrigel dome cultures. **a** Minced normal breast tissues from three reductive mammoplasty samples were dissociated and used to derive matched Matrigel dome and suspension organoid cultures. Scheme was created with BioRender.com **b** Representative brightfield images of the three matched organoid lines grown as Matrigel domes (MD) or suspension cultures (SC) for 5 passages and their corresponding mean organoid size. Scale bar 200 μm. A total of 30 organoids per line per culture condition was measured using Image J. ***p value < 0.001, ****p value < 0.0001, paired t-Test, two-tailed. **c** Percentage of EdU-positive cells in the three organoid lines at passage 5. **d** Percentage of HS, LASP and BA cells based on FACS analyses of EpCAM/CD49f expression. **e** Percentage of EdU-positive cells in the indicated organoid lines 10 days after changing the culture condition from suspension to Matrigel domes (SC - > MD) or reseeding as suspension culture (SC - > SC).

To evaluate the two culture methods for establishment of organoids directly from breast tissues, we thawed frozen minced breast tissues from three donors as previously described^14^, dissociated them and seeded the small clusters in either Matrigel domes or 5% Matrigel suspension as described above. We used TryPLE to passage the organoids and to free the cultures of debris and contaminating stromal cells. We noted differences in the morphology and size of organoids in these three cultures through the early passages. Since suspension cultures reached confluency faster than dome cultures, we diluted the suspension organoids into extra wells (after ~2 wks) until the dome cultures reached confluency (after ~4 wks) to ensure that the passage number was identical for each culture condition (Fig. 2a). After five passages with TryPLE, we imaged both Matrigel domes and suspension cultures after 10 days. The 5% Matrigel suspension cultures contained more and larger organoids than those generated in domes (Fig. 2b). We assessed the proportion of proliferating cells using EdU labeling as described above. Organoids in the suspension cultures contained a larger proportion of proliferating cells than those in Matrigel domes (Fig. 2c). We further analyzed proliferation specifically within the three epithelial cell types by flow sorting the cells using antibodies to EpCAM and CD49f (Fig. 2d and Supplementary Fig. 2a). Although the proportion of all three mammary epithelial lineages was largely comparable between the culture conditions in the three organoid lines, the fraction of proliferating cells within each lineage was higher in the suspension cultures than the Matrigel domes. We validated this finding using Ki-67 staining of ORG6 at passage 5 (Supplementary Fig. 2b). The percentage of Ki-67^+^ cells was consistent with what we observed with EdU labeling and confirmed that the fraction of proliferative cells was higher in suspension than Matrigel dome cultures (average of 45.9% in SC vs 38.2% in MD, Supplementary Fig. 2c). Consistent with what we observed with established organoid cultures, the percentage of Ki-67^+^ cells correlated with organoid size only in suspension cultures (Supplementary Fig. 2d).

To evaluate whether the differences in organoid proliferation are reversible, we then performed a short-term culture condition switch by transferring half of organoids from six organoid lines in suspension cultures into Matrigel domes for 10 days before Click-iT EdU assays (Fig. 2e). In all six organoid lines, there was a reduction in proliferation after transfer to Matrigel domes (average of 42% vs 22%; *p*-value < 0.01, paired t-Test, two tailed) (Fig. 2e). Moreover, half the organoid lines contain a higher proportion of proliferating HS cells and all organoid lines have more proliferating LASP and BA cells in suspension cultures compared to Matrigel domes (Supplementary Fig. 2e).

### Organoids cultured in suspension preserve breast epithelial cell lineage fidelity

**Fig. 3:**
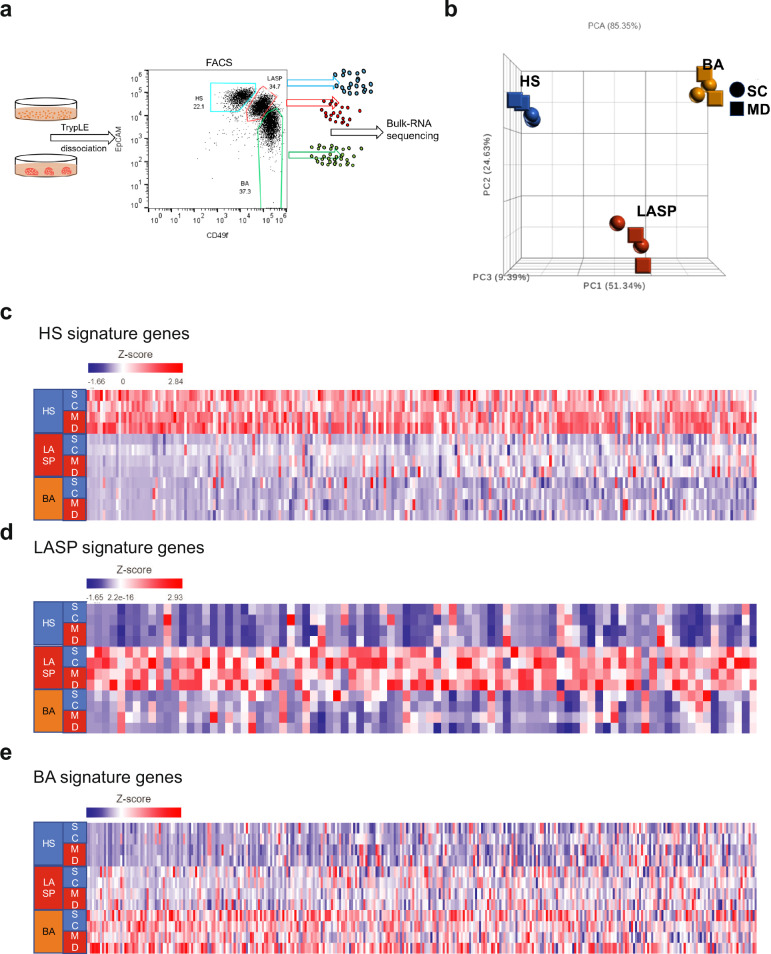
Organoid suspension cultures preserve breast epithelial lineage fidelity. **a** Organoid cultures were dissociated into single cells, FACS sorted into HS, LASP and BA cells based on differential expression of EpCAM/CD49f and total RNA was extracted for bulk RNA sequencing. **b** Principal Component Analysis (PCA) plot showing the HS, LASP and BA cell clusters isolated from organoids in Matrigel domes and suspension cultures. **c**–**e** Heatmaps showing the breast epithelial subtype-specific gene expression^15^ across the samples. (Gene list in Supplementary Table 2).

To assess the breast epithelial lineage fidelity in our organoid suspension cultures, we compared the gene expression profiles of EpCAM/CD49f-sorted HS, LASP, and BA cells from both dome and suspension organoids of ORG6 at passage 6–7 (Fig. 3a). RNA was extracted from the sorted cells and bulk sequenced (RNA-seq) to compare the gene expression patterns among the isolated cells. Principal Component Analysis (PCA) showed that the three breast lineage cell types clustered separately (Fig. 3b). We then examined the expression of breast lineage signatures defined from primary breast tissues by Gray et al.^15^. As shown in Fig. 3, the three breast epithelial lineages maintained their lineage fidelity in long-term organoid cultures (Fig. 3c–e). Among these three lineage cell types, HS cells preserved HS signature gene expression more faithfully than LASP and BA cells (Fig. 3c). In addition, LASP cells were found to express some HS and BA signature genes. The expression of these HS and BA genes could be due to cells undergoing differentiation from LASP to HS cells or BA cells. Previous reports indicated that luminal progenitors can generate both HS and BA like cells in vitro^6,13,16^.

In addition, the expression of HS and BA cell genes in LASP cells reflects the presence of the basal-luminal (BL) subtype of LASP cells which expresses markers of both BA and HS cells. We and others previously identified a subset of LASP cells that exhibit a reduction in lineage fidelity and express genes typically associated with BA or HS, as well as genes that are not enriched in any subtype of breast epithelial cells^15–21^. The BL signature is also associated with basal-like breast cancer based on identified BL-enriched genes that are poorly expressed in breast epithelial cells (BL-unique)^15^. Indeed, we confirmed that the BL-unique genes are highly enriched in the isolated LASP cells (Supplementary Fig. 3a). We also examined differentially expressed genes in each individual epithelial lineage from organoids cultured in either Matrigel domes or as suspension cultures. This analysis showed that only four HS signature genes, five LASP-specific genes (all higher in domes), and 23 BA-specific genes (12 genes expressed higher in suspension and 11 expressed higher in domes) were differentially expressed under these two conditions (Supplementary Fig. 3b). Overall, RNA-seq data indicates that organoids cultured under both conditions can preserve the lineage fidelity of breast epithelial cells.

### Hormone sensing cells can be isolated and propagated in the absence of other cell types

**Fig. 4:**
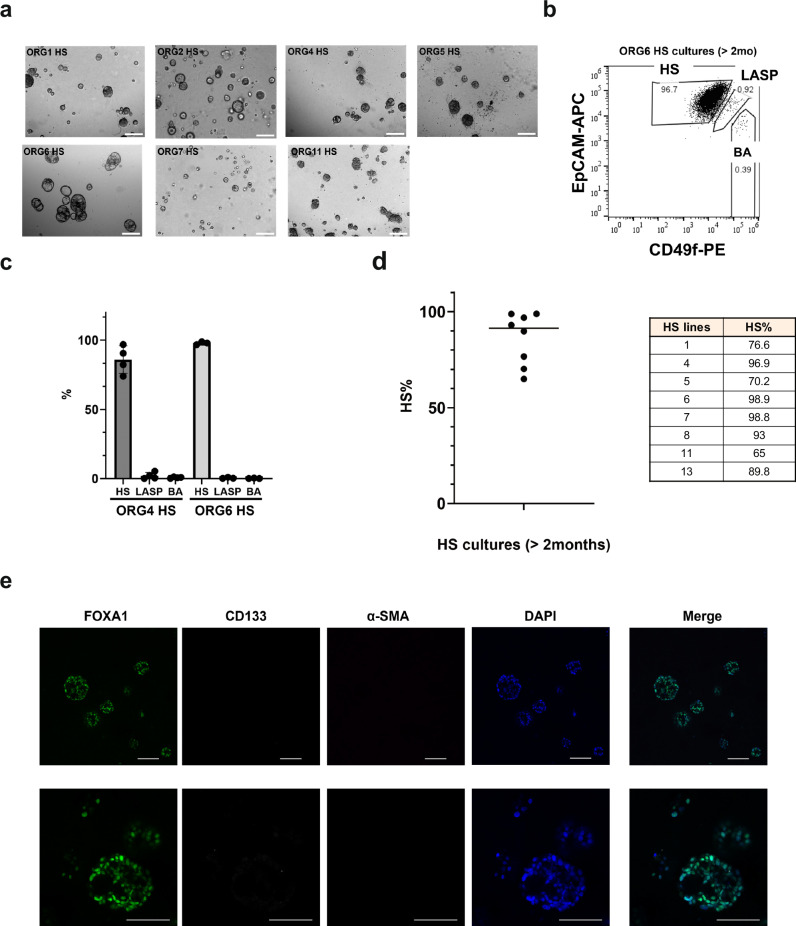
Hormone sensing cells can be isolated and propagated in suspension cultures in the absence of other cell types. **a** Representative brightfield images of four suspension cultures derived from sorted single HS cells based on expression of EpCAM/CD49f and cultured more than 2 weeks. 4× magnification, scale bar 200 μm. **b** Representative flow cytometry plot of HS cultures isolated from ORG6. **c** Percentage of HS, LASP and BA cells based on EpCAM/CD49f expression of HS cultures isolated from ORG4 (*n* = 4) and ORG6 (*n* = 3) and cultured for over 2 months, mean ± SD. **d** Percentage of HS cells based on expression EpCAM/CD49f in HS cultures isolated from eight different organoid lines and cultured for more than two months. **e** Representative immunofluorescence confocal images of HS cultures isolated from ORG2. Fluorescently conjugated antibodies targeting FOXA1, CD133 and α-SMA were used to detect the three different epithelial lineages and DAPI for nuclei staining. 20× magnification, scale bar 100 μm.

There is extensive crosstalk between the three subtypes of epithelial cells in vivo^22^. Since it would be useful to investigate and engineer HS cells independent of LASP and BA cells, we examined the properties of HS cells cultured alone. HS cells were isolated from seven different organoid lines by FACS using EpCAM and CD49f markers. To establish the HS cultures efficiently, we seeded EpCAM^high^/CD49f^low^ HS cells in suspension at high density (see Methods) in organoid culture medium supplemented with 5% Matrigel. All HS lines were able to form dense sphere-like or acinar-like structures within two weeks in suspension culture (Fig. 4a). We then examined the differentiation of HS-only cultures after long term passaging. After more than two months of suspension culture, we dissociated the organoids and performed flow cytometry analysis using EpCAM and CD49f to assess the relative proportion of epithelial cell types. Almost all organoid lines contained a high proportion of HS cells (EpCAM^high^/CD49f^low^), >70% in 7/8 lines and over 90% in 5/8 lines (Fig. 4b–d). To validate the flow cytometry result, we isolated HS cells from ORG2, cultured them separately in suspension for two months, and then immune-stained for canonical lineage markers (FOXA1 for HS, CD133 for LASP, and α-SMA for BA). The HS-only cultures were largely FOXA1^+^ (93% FOXA1^+^ cells per total nuclei) and negative for CD133 and α-SMA (Fig. 4e). To directly assess the lineage fidelity of HS-only cultures, we performed bulk RNA-seq of HS cells from ORG6 cultured alone for 7 days and compared HS signature gene expression to HS cells isolated directly from mixed lineage organoid culture (data from Fig. 3). We found that HS cells cultured alone clustered with HS cells directly isolated from organoids and maintained high HS signature gene expression compared to LASP and BA cells (Supplementary Fig. 4a, b). These results indicate that HS cells can be cultured in suspension in the absence of other cell types without significantly compromising their lineage fidelity.

### Organoids and HS cells cultured in suspension can activate ER signaling upon estrogen stimulation

**Fig. 5:**
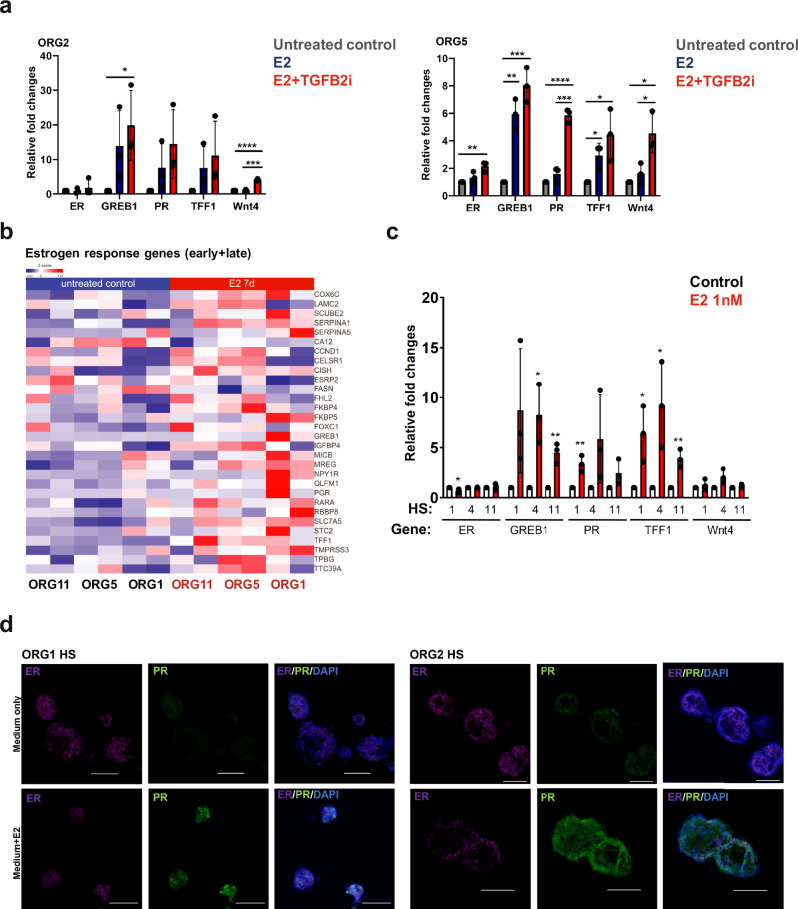
Organoids and HS cells cultured in suspension can activate ER signaling upon estrogen stimulation. **a** Expression of the indicated ER target genes in triplicate cultures from ORG2 and ORG5 suspension cultures after estrogen treatment for seven days, assessed by qRT-PCR (*n* = 3, mean ± SD). *p value < 0.05, **p value < 0.01, ***p value < 0.001, ****p value < 0.0001, paired t-Test, two-tailed. **b** Heatmap showing early and late estrogen response gene expression^30^ in HS cells isolated from three organoid lines in suspension cultures then treated with or without estrogen for seven days (duplicate) with *p* value < 0.05 as the cutoff. (Gene list in Supplementary Table 4). **c** Expression of the indicated ER target genes in triplicate HS cultures isolated from three organoid lines and treated with or without estrogen for seven days (*n* = 3, mean ± SD), assessed by qRT-PCR. *p value < 0.05, **p value < 0.01, paired t-Test, two-tailed. **d** Representative immunofluorescence confocal images of HS cultures from ORG1 and ORG2 treated with or without estrogen (E2) and stained for estrogen receptor (ER) and progesterone receptor (PR), scale bar 100 μm.

To assess if the HS cells in organoid suspension cultures still express ER and activate ER downstream signals upon estrogen stimulation after long-term culture (>2 months), we first treated different organoid lines (passage 6–9) with physiological concentrations of estrogen (E2, 1 nM) and progesterone (P4, 50 nM). We used quantitative reverse transcription-PCR (qRT–PCR) to measure changes in the expression of ER signaling genes such as *PR*, *TFF1* and *GREB1*^23–25^ as well as *WNT4*, a key canonical paracrine signaling protein regulated by PR in HS cells^26,27^. E + P treatment for seven days induced *TFF1*, *GREB1*, *PR* and *WNT4* in both organoids. TGFβ has been shown to suppress expression of ER and proliferation of HS cells^28^. While the organoid culture medium used in our experiments contains a TGFβ inhibitor (A83-01)^15^, we examined the effects of more effective TGFβ inhibition since it has been shown that supplementation with additional TGFβ inhibitors (SB431542 and RepSox) in cultures can increase ER expression and promote the growth of ER^+^ epithelial cells^28,29^. We found that of addition of RepSox and SB431542 increased the induction of ER downstream genes upon estrogen treatment (1.4-1.8-fold change in ORG2 and 1.4- to 3.7-fold in ORG5), without affecting ER expression in our organoid cultures. Moreover, induction of the PR-regulated gene *WNT4* was increased with additional TGFβ inhibition (2.8 to 4-fold across all organoid lines) (Fig. 5a and Supplementary Fig. 5a). Overall, we confirmed that ER and ER responses can be preserved in long-term organoid suspension cultures.

To measure the ER responses in isolated HS cells, we isolated HS from three organoid lines by FACS and kept them in suspension cultures supplemented with estrogen for 7 days. RNA was extracted and sequenced. The PCA plots indicated the heterogeneity of different samples, even for those originally derived from the same organoids (Supplementary Fig. 5b). However, there were many significantly upregulated or downregulated genes following treatment with estrogen (Supplementary Fig. 5c). In addition, we detected upregulation of many estrogen response genes^30^ in estrogen-treated HS cells (Fig. 5b). To address whether long-term culture of HS cells can also preserve ER expression as well as ER responses, we performed qRT-PCR to measure changes in expression of ER signaling related genes of three HS lines after being cultured for more than two months with or without seven days estrogen treatment. qRT-PCR results show that all three HS lines can still respond to estrogen stimulation (Fig. 5c). Furthermore, immunofluorescence staining for ER and PR in five HS lines cultured for more than two months confirms ER positivity (Fig. 5d) within HS cells and high PR expression upon ER stimulation (Fig. 5d and Supplementary Fig. 5d).

To evaluate whether ER expression and ER responses are similiar in the two different conditions, we cultured isolated HS cells from two organoid lines (ORG6 and ORG11) in suspension cultures and Matrigel domes separately for 10 days, then supplemented with estrogen for 7 days. Target gene expression was evaluated by qRT-PCR. There were no consistent differences in induction of ER-target genes in Matrigel domes compared to suspension cultures across the two HS lines during this time frame (Supplementary Fig. 5e).

Overall, our findings indicate that organoids and HS-only suspension cultures maintain ER expression and ER signaling and can be used as tools to study ER-related regulation in normal breast epithelial cells.

## Discussion

Organoids have been used extensively as in vitro models to investigate the development and physiology of normal organs and diseases including cancer. However, improvements to current breast organoid culture methodologies are needed to improve their utility. Here, we describe a modified organoid culture system for more effective expansion of normal breast organoids that maintain bilayered organoid morphology, lineage fidelity, and hormone receptor expression and responsiveness. As a result, our method represents a powerful tool to study estrogen and estrogen receptor regulation in normal tissues and ER positive breast cancer.

The conventional organoid culture system embedding organoids inside Matrigel domes was designed to recapitulate the in vivo extracellular matrix environment. However, passage of conventional dome cultures requires steps to break down Matrigel mechanically or enzymatically to separate organoids from mixtures. This physical stress could negatively affect organoid growth rate because organoids need more time to recover and reorganize following the harsher dissociation steps^10,12,31–33^. Moreover, the solid Matrigel dome culture system for normal breast organoids poses many challenges, such as requiring tedious steps for organoid propagation and failure to maintain bilayered organoid structures over time^12,14,34^. Our method using organoid culture medium supplemented with a low percentage of Matrigel (5%) simplifies the culture procedures for easier handling while still providing the sufficient extracellular matrix for organoid formation. Although the ratio of three epithelial lineages (HS, LASP and BA) was not significantly distinguished in dome or suspension cultures, organoids formed in suspension were larger and formed more uniform sphere structures than those in Matrigel domes. The structure size correlated with the proportion of proliferating cells in suspension cultures (Fig. 2c, e, Supplementary Fig. 1c–e, Supplementary Fig. 2b, c). It is also possible that suspension conditions allow cells to more efficiently migrate and aggregate compared to dome embedding. The maintenance of a bilayered morphology in suspension culture, with BA cells surrounding the luminal cells, could also contribute to the more uniform sphere structures (Supplementary Fig. 1g).

Bulk RNA seq analysis of the three breast epithelial cell types indicated that these three lineages maintain their cell identities in both types of organoid cultures. We detected minimal differences in cell identity signature genes in organoids cultured in domes or suspension. HS cells isolated from organoids in both culture conditions displayed higher lineage fidelity than LASP and BA cells (Fig. 3a). LASP cell populations expressed a few HS and BA genes; however, this is predicted based on the known presence of cell populations that display reduced lineage fidelity in human breasts. These cells, referred to as basal-luminal or BL cells, are enriched for genes specifically associated with BA or HS cells as well as genes that are expressed at very low levels or not at all in breast epithelial cells (BL-unique genes)^15,20,21^. The heatmap of BL-unique signature genes in Supplementary Fig. 3a indicates that the organoid cultures maintain BL cells. Thus, some of the BA (*KRT17, PTN, SPP1*) and HS (*FAM102A, HIGD1A, WFDC2*) genes that are expressed in LASP cells are likely due to the presence of a BL cell population in this cluster. Other BA genes expressed in LASP cells could be derived from BA cells that have undergone partial differentiation, sufficient for these cells to be sorted with LASP cells (e.g. decreased CD49f and increased EpCAM). Likewise, BA cells expressing LASP markers could have undergone a partial differentiation to LASP cells, without affecting CD49f/EpCAM sorting. BA cells have been reported to express LASP markers after being dissociated from luminal cells which are critical to maintain BA cell identity^6,35,36^.

Importantly, we were able to maintain purified HS cell organoids in suspension cultures while still maintaining extremely high purity after short-term (Supplementary Fig. 4a) or long-term culture (Fig. 4b–d). For a few HS organoid cultures, we did detect small percentages of LASP and BA cells. This could result from insufficient gating of HS cells during FACS using CD49f and EpCAM. Use of an additional sorting step or inclusion of more surface markers such CD166 for HS cells^17,35^, CD133 for LASP cells^15,37^ and CD10 for BA cells^38^ could also increase the purity of HS cells. Overall, we confirmed that HS cells can be isolated and cultured alone without losing cell identity (Supplementary Fig. 4a, b).

We found that the purified HS cells maintain expression of ER and respond to estrogen stimulation after long-term culture (Fig. 5c). We did not observe any consistent differences in ER-target gene expression comparing HS cells cultured in Matrigel domes to suspension cultures, suggesting that ER expression and ER responses are not influenced by the Matrigel conditions during the time frame we examined, and vary predominantly by donor (Supplementary Fig. 5e). Progesterone receptor induction of its target gene *WNT4* was only detected when organoids were cultured with additional TGFβ inhibitors (Supplementary Fig. 5a), consistent with previous studies using other medium conditions^28,29^.

One issue that all breast organoid investigations have reported is the heterogeneity of organoids from different donors. As observed previously^12,14^, we detected widely varying proportions of the three main cell lineages in organoid cultures from different donors. There are several factors that may account for this variation, such as age, menopause status, BMI, or genetic background (e.g., BRCA mutations). This heterogeneity makes it very difficult to identify phenotypic differences in organoids that can be attributed to the factors mentioned above. However, the proportions of each lineage from a single organoid line are maintained for several passages^12,14^, thus making it feasible to use genetic or pharmacological perturbations within the same organoid lines to address mechanistic questions.

In conclusion, our results demonstrate that organoid suspension cultures represent a valuable in vitro platform to study ER and estrogen regulation in HS cells from normal breast, enabling studies of ER-positive breast cancer initiation that were not previously feasible.

## Methods

### Generation of organoids from breast tissues

Breast tissues were obtained from reduction mammoplasty or prophylactic mastectomy samples at Brigham & Women’s Hospital. Harvard Medical School Institutional Review Board reviewed this study and deemed it not human subject research. Donors gave informed consent to have their tissue used for research purposes. Tissues were processed as previously described^14^. Briefly, tissues were processed on the day of surgery by mincing into small chunks. Minced tissue was placed into a conical tube containing triple+ Adv medium (Advanced DMEM/F12 supplemented with 1× Glutamax, 10 mM HEPES, and Pen-Strep) and 1 mg/ml collagenase (Sigma, C9407) for tissue dissociation. Some of the viable minced tissue was also frozen (in FBS with 10% DMSO) for future use. Tubes were placed in the orbital shaker at 37 °C for 2 h. After collagenase dissociation, triple+ Adv medium with 2% FBS was added before centrifugation. The dissociated tissue pellets were resuspended in 10 ml triple+ Adv medium and underwent further mechanical shearing into small clusters of 20–60 cells by sequential pipetting with 10 and 5 ml serological pipettes. Supplementary Table 1 provides information on the donor tissues employed in this study.

### Organoid cultures

Organoids were cultured as previously described^12,14^. For Matrigel dome cultures, primary breast organoids were resuspended in Matrigel growth factor reduced (GFR) basement membrane matrix (Corning, cat. No. 354230) and dropped in the center of a well in a 24-well culture plate (Corning, cat. No. 3524) and placed at 37 °C incubator for 10–20 min to form the solid domes before adding organoid culture medium (Supplementary Note 1). Medium was changed twice per week, and organoids were passaged using TrypLE™ Express Enzyme (1X), no phenol red (Gibco, cat. No. 12604013) to dissociate into small clusters when organoids reached 80% confluency in the domes (about every 2–4 weeks). For suspension organoid cultures, primary breast organoids were resuspended in organoid culture medium containing 5% Matrigel and cultured in Costar® 24-well ultra-low attachment plates (Corning, cat. No. 3473). Suspension cultures were supplemented with an equal amount of fresh organoid culture medium containing 5% Matrigel every 3–4 days and transfered to larger culture wells to maintain 40–70% confluency. When most organoids reached 100–200 μm size, we passaged them by using TrypLE™ Express Enzyme (1X), no phenol red (Gibco, cat. No. 12604013) to dissociate into small clusters (about every 2–4 weeks). Dissociated small organoid clusters were collected into 15 ml conical tubes and centrifuged at 500rcf for 4 min. Supernatants were removed and the organoid pellets were resuspended with organoid culture medium containing 5% Matrigel. For seeding of HS-only cultures, we seeded EpCAM^high^/CD49f^low^ HS cells in suspension at a density of 40,000–50,000 cells in 500 μl organoid culture medium supplemented with 5% Matrigel.

### Hormone stimulation

Cultures were treated for seven days with 1 nM beta-estradiol in organoid culture medium added on days 1, 4 and 7, and 50 nM progesterone added at day seven for 7 h before RNA extraction.

### Flow cytometry and Click-iT EdU assay

Organoid cultures were treated with 5-ethynyl-2’-deoxyuridine (EdU) at 10 µM for 48 h, then dissociated to single cells by TrypLE express enzyme. Cells were fixed and labeled using the Click-iT™ Plus EdU Alexa Fluor™ 488 Flow Cytometry Assay Kit (Invitrogen, Cat. No. C10632) following the manufacturer’s protocol combined with the breast epithelial lineage markers Alexa Fluor 647-conjugated anti-EpCAM (BioLegend, Cat. No. 324212) (1:100) and phycoerythrin-conjugated anti-CD49f (BioLegend, Cat. No. 313612) (1:100). Flow cytometry data were analyzed with FlowJo software.

### Fluorescence-activated cell sorting (FACS) isolation of mammary epithelial lineage cells

Organoid cultures were dissociated into single cells as described above and labeled with Alexa Fluor 647-conjugated anti-EpCAM (1:100) and phycoerythrin-conjugated anti-CD49f (1:100) in staining buffer (dPBS contained 2% FBS and 20 mM HEPES) for 1 h at room temperature. EpCAM^high^ CD49f^low^ HS cells, EpCAM^high^ CD49f^high^ LASP cells, and EpCAM^low^ CD49f^high^ BA cells were sorted on a SONY SH800S Cell Sorter. Sorted cells were cultured in organoid culture medium with 5% Matrigel or immediately used for RNA extraction. Flow cytometry data were analyzed with FlowJo software.

### Immunofluorescence (IF)

Immunofluorescence performed as described previously^39^ with minor modifications depending on the specific antibodies. Cultured organoids or cells were transferred onto Falcon® 8-well Culture Slides (Corning, cat. No. 354118) and fixed in 4% formaldehyde for 15 min. Samples were permeabilized in 0.5% Triton X in dPBS for 15 min and then blocked for 1 h in 1% BSA in IF solution (dPBS with 0.2% Triton X and 0.05% Tween 20). For unconjugated primary antibodies, samples were incubated at the appropriate concentration (1:100 for CD133, 1:150 for α-SMA) of primary antibody overnight at 4 °C and incubated in diluted (1:200–1:400) secondary antibody conjugated with fluorophores for 1 h at room temperature. For direct conjugated primary antibodies, samples were incubated in the appropriate concentration (1:100 for ERα, 1:150 for PR, 1:200 for FOXA1) of primary antibody conjugated with fluorophores overnight at 4 °C. Nuclei were stained with DAPI. After staining, slides were mounted in ProLong™ Gold Antifade Mountant (Invitrogen, Cat. No. P36930) before detection. Stained samples were imaged on the Nikon A1R point scanning confocal microscope or Nikon AX-R point scanning confocal microscope. Images were processed and analyzed using NIS-Elements Viewer and ImageJ software.

### RNA isolation

Total RNA was extracted using TRIzol/Chloroform method according to the manufacturer’s instructions with nuclease-free reagents. For organoids in 24-well plates, 1 ml TRIzol per well was used for the RNA extraction and a ratio of 200 µl chloroform to 1 ml of TRIzol reagent for separation. RNA was precipitated from the aqueous phase with isopropyl alcohol at a ratio 0.5 ml to 1 ml of TRIzol and 1 µl GlycoBlue™ Coprecipitant (Invitrogen, Cat. No. AM9516). The RNA pellet was washed with 75% ethanol once, air-dried and resuspended in 10 µl nuclease-free water (Invitrogen, Cat. No. AM9932). RNA concentration was measured by A260/A230 and A260/A280 ratios on a Nanodrop One. RNA samples were used for reverse transcription and quantitative real-time PCR or RNA sequencing.

### Reverse transcription and quantitative real-time PCR (qRT-PCR)

1 μg of total RNA per sample was used for reverse transcription. After DNase I digestion, RNA samples were reverse transcribed into cDNA using TaqMan Reverse Transcription Reagent kit (Invitrogen, Cat. No. N8080234) according to manufacturer’s instructions. cDNA samples mixed with primer sets and Power SYBR Green PCR Master Mix (Applied Biosystems™, Cat. No. 4367659) were used for qRT-PCR on an Applied Biosystems QuantStudio 7 Pro machine. Human *RPS28* and *RPL13A* were used as endogenous controls to normalize each sample. Reagents and primer sequences used in this study are provided in Supplementary Note 2.

### RNA sequencing (RNA-seq) and data analysis

RNA samples were sent to Novogen for library preparation and mRNA sequencing using Illumina NovaSeq 6000 and X-Plus Sequencing Platform (paired-end 150 bp). Data were analyzed using Partek Flow software. Briefly, reads were mapped to hg38 using STAR2.7.8a then quantified to annotation model by Partek E/M (GENCODE genes version 38). Differential gene expression was performed with DESeq2, and FDR < 0.1 or *p*-value < 0.05 was used as a threshold for statistical significance. Heatmaps were generated by applying signature gene lists^15,30^.

### Quantification and statistical analysis

Statistical tests were performed and analyzed using Microsoft Excel and GraphPad Prism 10 with *p* value analysis setting (paired t-Test, two-tailed). Significance was defined as *p* < 0.05.

## Supplementary information

Supplementary Information

Supplementary Table 2

Supplementary Table 3

Supplementary Table 4

Supplementary Table 5

## Source data

Source data

## Data Availability

The raw and processed bulk RNAseq data have been deposited in Gene Expression Omnibus (GEO): GSE266935. All the other data supporting the findings of this study are available within the article and its Supplementary Information files and from the corresponding author upon reasonable request.

## Code availability

The RNA-seq analyses and heatmaps were performed by using Partek Flow software. No code was written for the study.
