# Supplementary material for: Breast organoid suspension cultures maintain long-term estrogen receptor expression and responsiveness
Source: NPJ Breast Cancer. 2024 Dec 19;10:107. doi: 10.1038/s41523-024-00714-7 (PMC11659324; doi:10.1038/s41523-024-00714-7)
Supplement: Supplementary file 1 — Supplementary Information [file 41523_2024_714_MOESM1_ESM.pdf]

Supplementary Figure 1

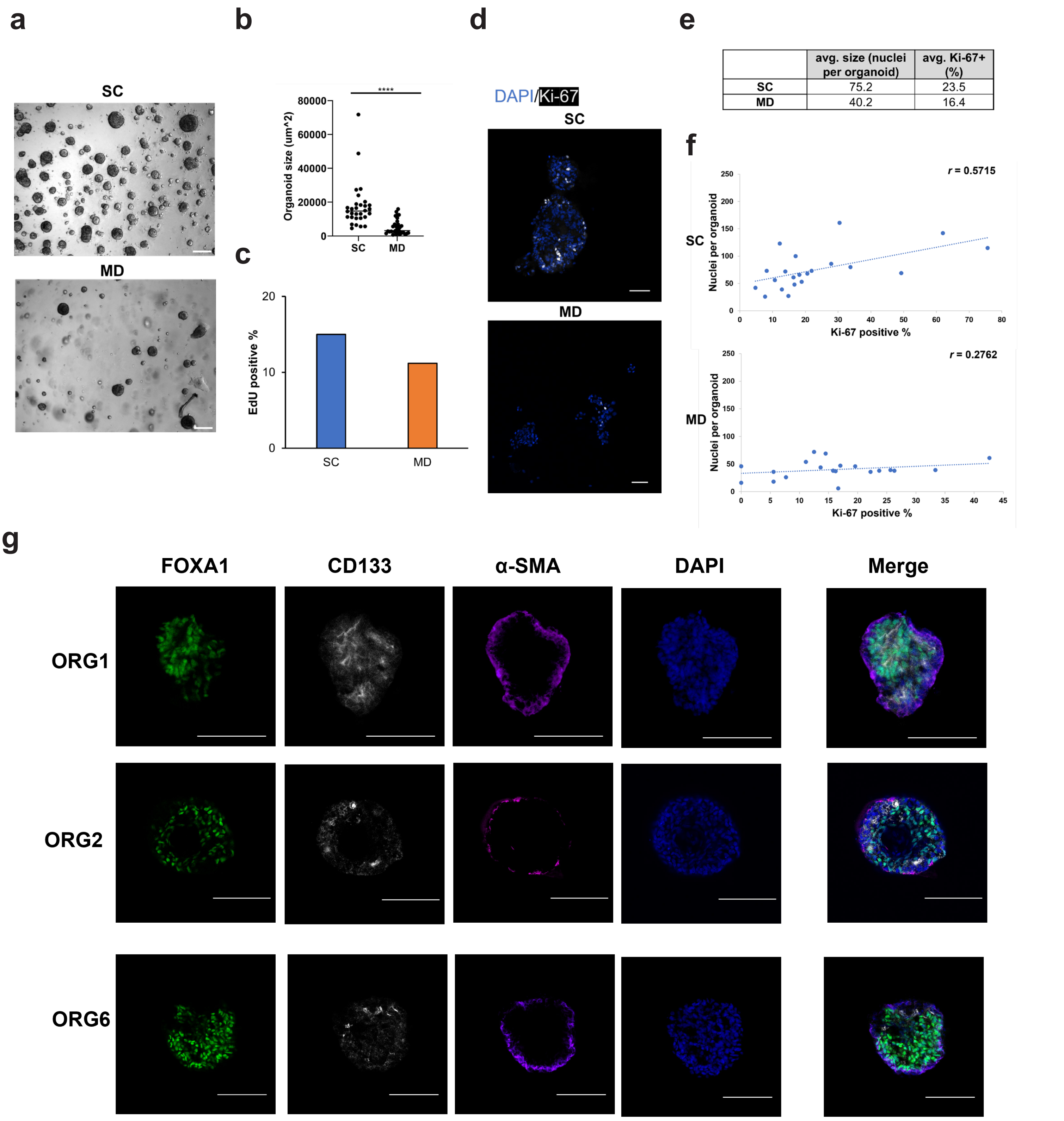

	avg. size (nuclei per organoid)	avg. Ki-67+ (%)
SC	75.2	23.5
MD	40.2	16.4

Characterization of organoid cultures. a) Established breast organoid cultures (ORG14) derived using the conventional Matrigel dome method (passage 5-6[14]) were dissociated and either re-cultured in Matrigel domes or as suspension cultures in 5% Matrigel for more than two months. Representative brightfield images of ORG14 (4x magnification, scale bar 200 µm) and **b**) mean organoid size of the indicated matched cultures grown as domes or in suspension. A total of 30 organoids per line per culture condition was measured using Image J. **** p value <0.0001, paired t-Test, two-tailed. **c**) Percentage of EdU-positive cells in ORG14 organoids grown as Matrigel domes (MD) or suspension cultures (SC). **d**) Representative immunofluorescence confocal images of organoids stained with antibody to Ki-67 and DAPI (nuclei) that were used to quantitate % of proliferating cells in panels e,f. 20x magnification, scale bar 100 µm. **e**) 20 organoids (per condition) were counted for Ki-67 positivity and the average number of nuclei and %Ki-67+ cells was calculated. **f**) Pearson analysis was conducted to determine correlation coefficient (*r*) values, assessing the correlation between organoid size and the percentage of Ki-67 positive cells within the organoids. g) Representative immunofluorescence confocal images of organoids stained with antibodies to three epithelial lineage markers FOXA1 (hormone sensing cells/HS), CD133 (luminal adaptive secretory precursor/LASP), and α-SMA (basal cells/BA) in organoids cultured in suspension cultures. DAPI was used to stain nuclei. 20x magnification, scale bar 100 µm.

Supplementary Figure 2

a

EdU positive %	HS cells		LASP cells		BA cells	
**Organoid lines**	**SC**	**MD**	**SC**	**MD**	**SC**	**MD**
ORG12	9.6	7.1	47	45	39.3	30.1
ORG5	15.8	2.7	46.6	18	31.2	7.35
ORG6	56.1	46	57	53	34.2	26.6

b

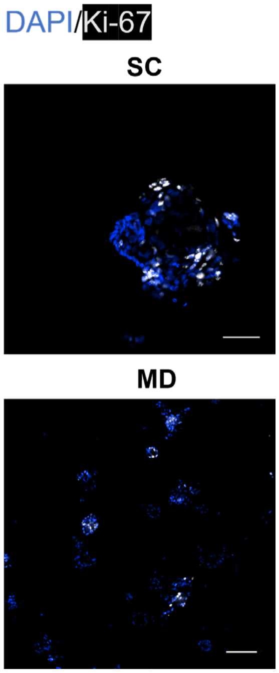

c

	avg. size (nuclei per organoid)	avg. Ki-67+ (%)
**SC**	96.9	45.9
**MD**	26.1	38.2

d

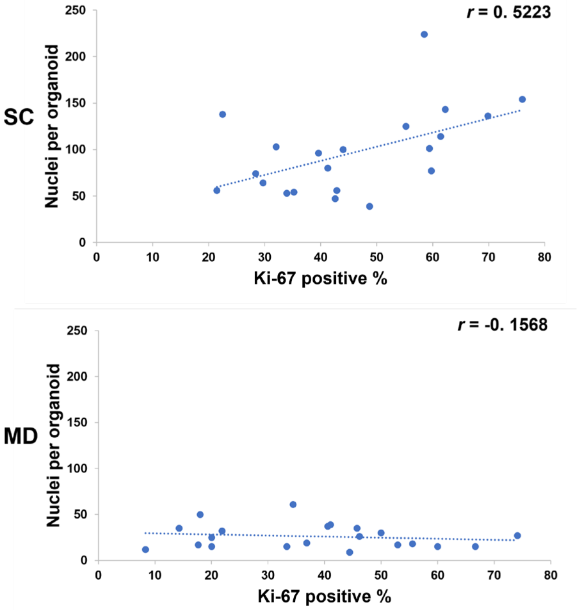

e

EdU positive %	HS cells		LASP cells		BA cells	
**Organoid lines**	**SC > SC**	**SC > MD**	**SC > SC**	**SC > MD**	**SC > SC**	**SC > MD**
2	13.8	13.5	26.6	11	56.8	20.3
5	36.5	29.4	54.1	24.3	56.4	21.5
7	30.5	20	41.2	7.6	42.1	10.9
8	23.4	28.4	51.5	23.1	40.5	20.3
9	23.5	23.4	66.6	36.8	65.8	34.2
11	37.6	16.3	55.3	44.1	55.3	44.1

Proliferation analysis of epithelial lineages within organoids cultured in Matrigel domes or suspension. **a**) Percentage of EdU-positive cells in HS, LASP, and BA cells sorted based on EpCAM/CD49f from three organoid lines (ORG12, ORG5 and ORG6; passage 5) cultured in conventional Matrigel domes (MD) or suspension cultures (SC). **b**) Representative immunofluorescence confocal images of cells stained with antibodies against Ki-67 and DAPI was used for nuclei staining in ORG6. 20x magnification, scale bar 100 μm. **c**) 20 organoids per condition from ORG6 were counted for total nuclei and Ki-67 positive nuclei and **d**) Pearson analysis was conducted to determine correlation coefficient (*r*) values, assessing the correlation between organoid size and Ki-67 positive % in organoids. **e**) Percentage of EdU-positive cells in HS, LASP, and BA cells sorted based on EpCAM/CD49f from six organoid lines (ORG2, ORG5, ORG7, ORG8, ORG9, ORG11) 10 days after changing the culture condition from suspension cultures to Matrigel domes (SC -> MD) or reseeding as suspension culture (SC -> SC).

Supplementary Figure 3

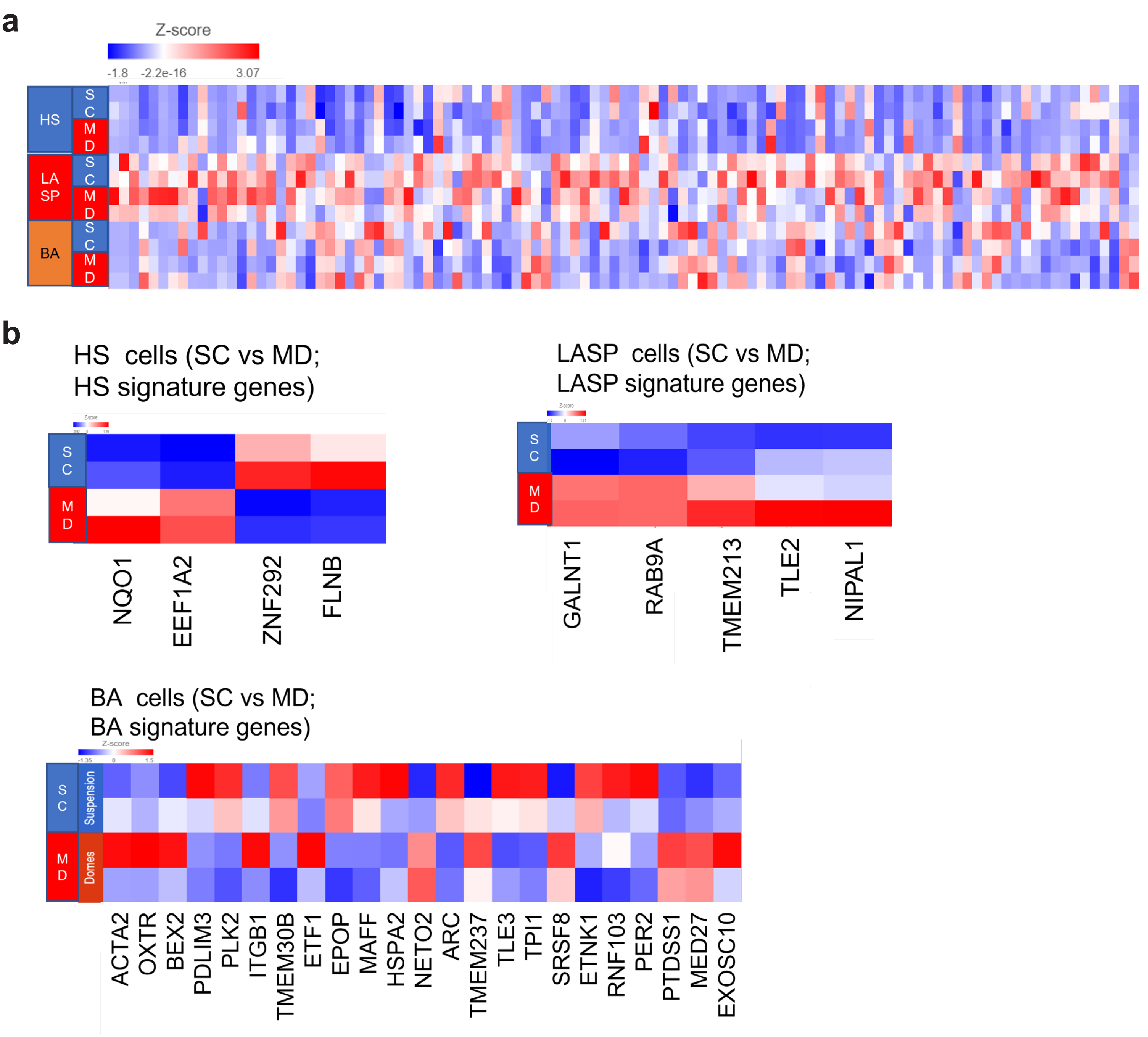

Heatmaps of the gene expression of the **a**) BL unique signature (gene list in Supplementary Table 2), **b**) differential expressed genes analyses of HS signatureHS signature, LASP signature, and BA signature from the indicated cell types sorted from either Matrigel domes (MD) or suspension cultures (SC) based on EpCAM/CD49f expression with p value <0.05 and FDR <0.1 as the cutoff. Signatures from[15].

Supplementary Figure 4

a

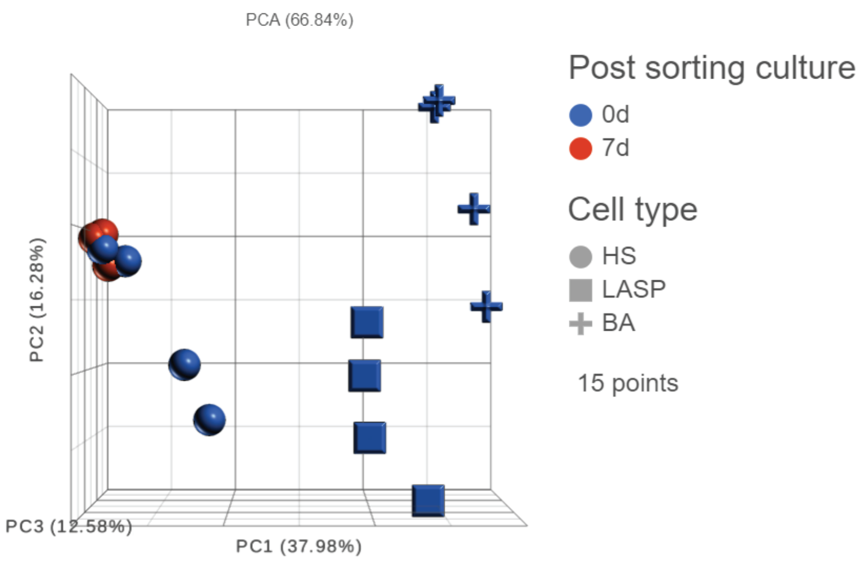

b

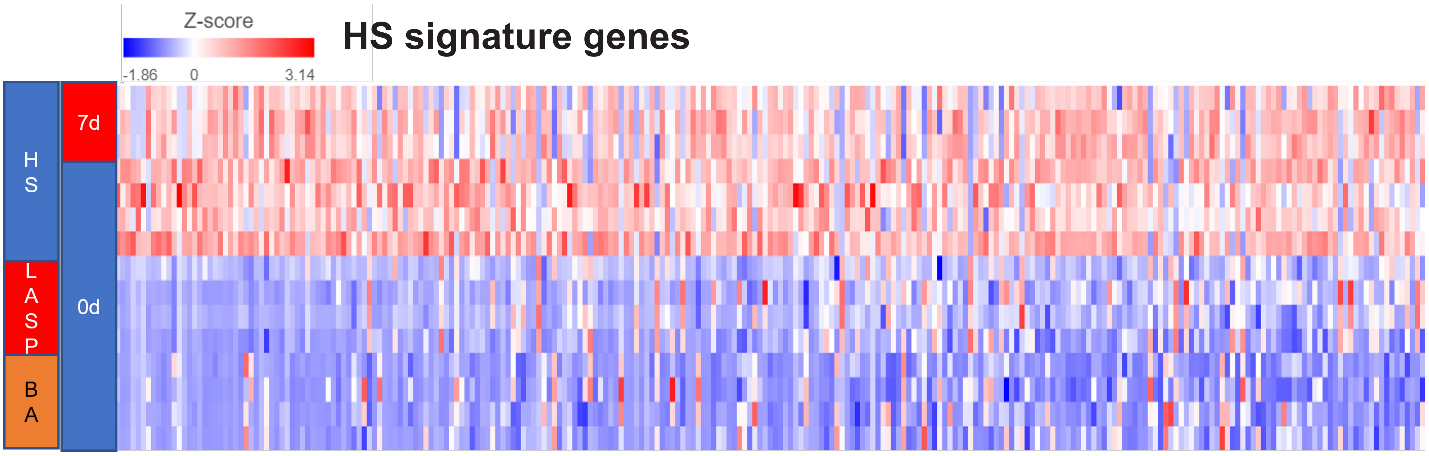

a) Principal Component Analysis (PCA) plot showing the HS, LASP and BA cell clusters isolated from ORG6 in suspension cultures. HS cells were collected right after isolated from organoids (0d, n=4) or cultured alone for 7 days (7d, n=3) for RNA-seq. LASP (n=4) and BA cells (n=4) were collected right after isolated from ORG6 for RNA-seq. **b**) Heatmaps showing the HS specific gene expression[15] across the samples. (Gene list in Supplementary Table 3)

Supplementary Figure 5

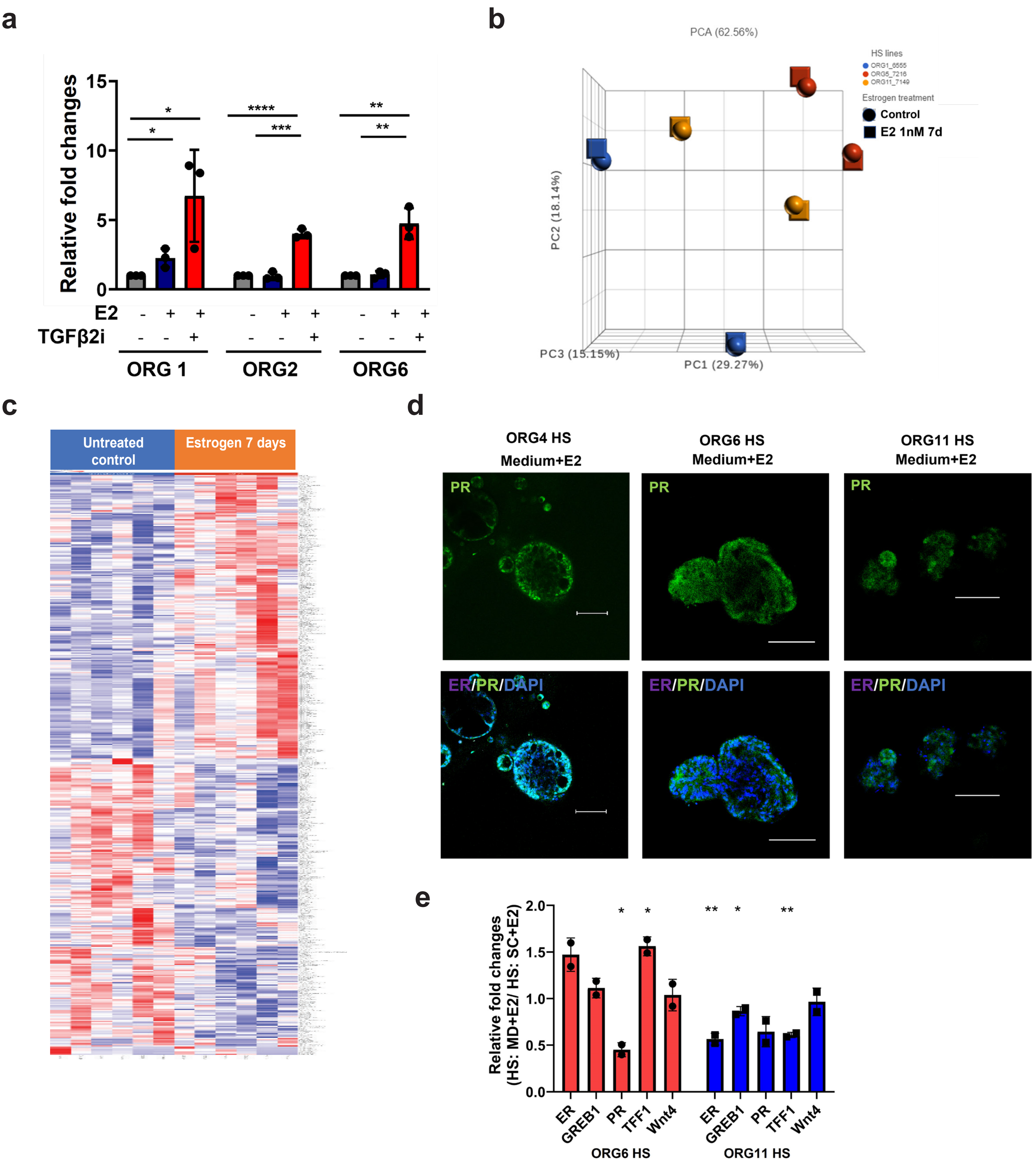

a) Wnt4 gene expression in the indicated organoid lines after estrogen treatment for 7d with or without additional TGFβ2i (RepSox and SB431542) assessed by qRT-PCR (n=3, mean±SD). * p value <0.05, ** p value <0.01, *** p value <0.001, **** p value <0.0001, paired t-Test, two tailed. **b**) PCA plot showing the clusters of three HS lines with or without estrogen treatment for 7d. **c**) Heatmap showing genes differentially expressed between untreated controls and organoids treated with estrogen for 7d in three different HS lines with p value <0.05 as the cutoff. (Gene list in Supplementary Table 5) **d**) Representative immunofluorescence confocal images of HS cultures from ORG4, ORG6, and ORG11 treated with estrogen (E2) and stained for estrogen receptor (ER) and progesterone receptor (PR), scale bar 100 µm. **e**) ORG6 and ORG11 sorted HS cells were cultured in Matrigel domes (MD) or suspension cultures (SC) separately for 10 days then supplemented with or without estrogen for 7 days. Expression of ER and the indicated ER target genes in HS cultures was assessed by qRT-PCR. (n=2, mean±SD). * p value <0.05, ** p value <0.01, paired t-Test, two tailed.

Supplementary Table 1: Tissue donor information of organoids used in this study List of assays and resources used in this study

No.	Age	Gender	Race	Gene	Alteration	GT result	Menopause Status
ORG1	48	Female	White or Caucasian	Normal			Premenopause
ORG2	21	Female	White or Caucasian	Normal			Premenopause
ORG3	28	Female	White or Caucasian	Normal			Premenopause
ORG4	58	Female	White or Caucasian	BRCA2	c.8537_8538delAG (p.Glu2846Glyfs*22)	Pathogenic	Surgical Menopause
ORG5	67	Female	White or Caucasian	Normal			Postmenopause
ORG6	47	Female	Asian	Normal			Premenopause
ORG7	24	Female	White or Caucasian	Normal			Premenopause
ORG8	25	Female	White or Caucasian	BRCA1	EX13_18del	Pathogenic	Premenopause
ORG9	65	Female	White or Caucasian	BRCA1	c.5266dupC	Pathogenic	Postmenopause
ORG10	48	Female	White or Caucasian	BRCA2	c.4631del (p.Asn1544Thrfs*24)	Pathogenic	Perimenopause
ORG11	30	Female	White or Caucasian	Normal			Premenopause
ORG12	38	Female	White or Caucasian	Normal			Premenopause
ORG13	35	Female	White or Caucasian	BRCA2	c.7007+5G>A	Pathogenic	Premenopause
ORG14	45	Female	White or Caucasian	Normal			Surgical Menopause

Supplementary Note 1 : Reagents and components used for organoid culture medium and this study

No.	Ingredient	Resuspension buffer	Stock conc	Final conc	Stock to final conc. Ratio	add to 50mL (uL)	Vendor	Cat#
1	R-spondinl	0.1%BSA/PBS	100 ug/mL	200 ng/mL	1:500	100	MedChemExpress (MCE)	120-38-50UG
2	Noggin	0.1%BSA/PBS	100 ug/mL	100 ng/mL	1:1000	50	MedChemExpress (MCE)	HY-P7051A
3	B27 supplement		50x	1x	1:50	1000	Life Tech	17504044
4	N-acetyl L-cysteine	H2O	1 M	1.25 mM	1:800	62.5	Sigma	A9165-5G
5	Nicotinamide	PBS	1M	10mM	1:100	500	Sigma	N0636-100G
6	Y-27632 (ROCKi)	H2O	10 mM	5 uM	1:2000	25	ENZO	ALX-270-333-M025
7	A83-01 (ALKi)	DMSO	500 uM	500nM	1:1000	50	Tocris	2939-10mg
8	SB202190 (p38i)	DMSO	10 mM	1 uM	1:10000	5	Sigma	S7067-5MG
9	h FGF-7	0.1%BSA/PBS	10 ug/mL	5 ng/mL	1:2000	25	Peprotech	100-19-50UG
10	h FGF-10	0.1%BSA/PBS	100 ug/mL	20 ng/mL	1:5000	10	Peprotech	100-26- 50UG
11	HeregulinB1	0.1%BSA/PBS	10 uM	5 nM	1:2000	25	Peprotech	100-03-50UG
12	hEGF	0.1%BSA/PBS	100 ug/mL	5 ng/mL	1:20000	2.5	Peprotech	AF-100-15
13	Adv DMEM/F12					46645	Invitrogen	12634028
14	GlutaMax 100x					500	Invitrogen	35050-061
15	HEPES 1M			10mM		500	Invitrogen	15630-080
16	Pen/Strep					500	Gibco	15070063

REAGENT	SOURCE	IDENTIFIER
Chemicals, enzymes		
TRIzol Reagent	Life Technologies	Cat# 15596018
Chloroform	VWR	Cat# MK443210
Isopropanol	ThermoFisher	Cat# BP26184
Dnase I	ThermoFisher	Cat# 18068015
Paraformaldehyde	Electron Microscopy Sciences	Cat# 19002
Triton X-100	Sigma-Aldrich	Cat# T8787
Bovine Serum Albumin	Sigma-Aldrich	Cat# A2153
DMSO	Sigma-Aldrich	Cat# D2650
Tween-20	Sigma-Aldrich	Cat# P1379
GlycoBlue	Life Technologies	Cat# AM9516
ProLong Gold Antifade Mountant	Life Technologies	Cat# P36930
DAPI	ThermoFisher	Cat# D1306
Matrigel (growth factor reduced)	Corning	Cat# 354230
Cultrex Reduced Growth Factor BME, Type 2, Pathclear	Cultrex	Cat#3533-010-02
TrypLE™ Express Enzyme (1X), no phenol red	Gibco	Cat#12604013
Collagenase from Clostridium histolyticum	Sigma-Aldrich	Cat#C9407
B-Estradiol	Sigma-Aldrich	Cat#E2257
Progestrone	Sigma-Aldrich	Cat#P8783
SB431542	Selleck Chemicals	Cat#S1067
RepSox	Stemcell	Cat#73792

Supplementary Note 2: Primer sets for qRT-PCR and antibodies for IF and FACS in this study

Primer	Sequence :
h-ESR1-F	AAGAGCTGCCAGGCCTGCC
h-ESR1-R	TTGGCAGCTCTCATGTCTCC
h-PGR-F	GGGTGCTGGAGGCAGCAGTT
h-PGR-R	GGAGGGCTGGGTTGGCTCTG
h-TFF1-F	GGTGATCTGCGCCCTGGTCCT
h-TFF1-R	ACTGGGAGGGCGTGACACCA
h-GREB1-F	CGCCAGCTCCTGCAACGACA
h-GREB1-R	GCGGCAGGCGCAGATGATGA
h-WNT4-F	GCATCTCAGAGGAGGAGACG
h-WNT4-R	GGAACTGGTACTGGCACTCC
h-RPS28-F	CGATCCATCATCCGCAATG
h-RPS28-R	AGCCAAGCTCAGCGCAAC
h-RPL13A-F	CCTGGAGGAGAAGAGGAAAGAGAAA
h-RPL13A-R	TTGAGGACCTCTGTGTATTTGTCAA

REAGENT or RESOURCE	SOURCE	IDENTIFIER
Antibodies		
Estrogen Receptor α (D6R2W) Rabbit mAb (Alexa Fluor® 647 Conjugate)	Cell Signaling	Cat# 57761; RRID:AB 2799533
Progesterone Receptor A/B (D8Q2J) XP® Rabbit mAb (Alexa Fluor® 488 Conjugate)	Cell Signaling	Cat #35591; RRID:AB 2799080
Recombinant Alexa Fluor® 488 Anti-FOXA1 antibody [EPR10881]	abcam	Cat# ab197235, RRID:N/A
CD133 (D2V8Q) XP® Rabbit mAb	Cell Signaling	Cat# 64326; RRID: AB_2721172
α-Smooth Muscle Actin (D4K9N) XP® Rabbit mAb (Alexa Fluor® 647 Conjugate)	Cell Signaling	Cat# 76113; RRID: AB_2857972
Alexa Fluor® 568 Goat Anti-Rabbit IgG (H+L), highly cross-adsorbed	Invitrogen	Cat# ab129002; RRID: N/A
Alexa Fluor® 647 anti-human CD326 (EpCAM) Antibody	Biolegend	Cat# 324212; RRID:AB_756086
PE anti-human/mouse CD49f	Biolegend	Cat# 313612; RRID:AB_893374
Ki-67 Monoclonal Antibody (20Raj1), eFluor™ 570, eBioscience™	Invitrogen	Cat#41-5699-82; RRID:AB 11220278

Supplementary Note 3: List of assays and resources used in this study

ASSAY and RESOURCE	SOURCE	IDENTIFIER
**Critical Commercial Assays**		
PowerUp SYBR Green Master Mix	ThermoFisher	Cat# A25742
TaqMan Reverse Transcription Reagents	ThermoFisher	Cat# 4304134
**Deposited Data**		
RNA sequencing data	Gene Expression Omnibus	GSE266935
**Eperimental Models**		
Human breast tissues		
Donor infromation see supplementary data 1		
**Oligonucleotides**		
RT-PCR primer sequences see supplementary data 3	This paper	N/A
**Antibodies**		
List of Antibodies used for this study see supplementary data3	This paper	N/A
**Software and Algorisms**		
ImageJ	NIH	https://imagej.net/ij/
GraphPad Prism	GraphPas Software	https://www.graphpad.com/scientific-software/prism/
FlowJo	FLOWJO, LLC	https://www.flowjo.com/
Biorender	Biorender	https://www.biorender.com/
NIS-Elements Viewer	Nikon	https://www.microscope.healthcare.nikon.com/products/software/nis-elements/viewer
Partek Flow Genomic Analysis Software	Partek	https://www.partek.com/partek-flow/
